# Exploratory ferroptosis-related transcriptomic patterns in dilated cardiomyopathy and pan-cancer datasets with H9c2 RT-qPCR assessment

**DOI:** 10.3389/fgene.2026.1909716

**Published:** 2026-09-22

**Authors:** Mingwei Yang, Huayin Li, Fangchao Wang, Jiao Wang, Boli Ran, Rui He

**Affiliations:** Department of Cardiology, Chongqing General Hospital, Chongqing University, Chongqing, China

**Keywords:** bulk transcriptomics, dilated cardiomyopathy, ferroptosis, hypertensive heart disease, pan-cancer, RT-qPCR, single-cell RNA sequencing

## Abstract

**Background and Aims:**

Ferroptosis-related pathways have been implicated in cardiovascular remodeling and cancer, but their relevance to hypertensive heart disease (HHD) remains uncertain. This study examined transcriptomic patterns in a dilated cardiomyopathy (DCM) dataset and used pan-cancer data only for descriptive context; it did not analyze primary HHD tissue.

**Methods:**

GSE183852 data were restricted to the explicitly identified non-diseased sample H_ZC-11-292 (8,749 cells) and DCM sample HDCM1 (7,202 cells). H_ZC-LVAD-1 was excluded because GEO metadata classify it as DCM. Full-rank cell-level Wilcoxon results were retained, with all single-cell tests treated as descriptive. GSE5406 groups were assigned from GEO sample titles (194 failing and 16 non-failing myocardium samples), and TCGA data were used for descriptive pan-cancer context. GPX4, SLC7A11, ACSL4, and TFRC mRNA were assessed by RT-qPCR in Ang II-stimulated H9c2 cells.

**Results:**

Fourteen cluster-derived cell categories were retained. Cardiomyocytes, macrophages, and endothelial cells were not detected in the selected DCM donor, so no condition-specific inference was made for those categories. Among 109 detected ferroptosis-related genes, 60 met the cell-level up threshold, 24 met the down threshold, and 25 were nonsignificant; these counts are not donor-level evidence. In GSE5406, 5 of 99 tested ferroptosis-related genes met |log2FC| > 0.5 and BH-adjusted p < 0.05. Fold changes for 96 detected genes were uncorrelated across platforms (r = 0.034, p = 0.740). RT-qPCR showed lower GPX4 and SLC7A11 and higher ACSL4 and TFRC mRNA, with gene- and context-specific directionality (Supplementary Table S1).

**Conclusion:**

The analyses identify exploratory, donor- and context-dependent transcriptional patterns. They do not establish ferroptosis as a causal mechanism, biomarker, or treatment target in primary HHD. Multi-donor disease-specific cohorts and protein-level and functional experiments are required.

## 1 Introduction

Hypertensive heart disease (HHD) comprises progressive structural and functional cardiac changes associated with chronic pressure overload (Messerli et al., 2017). Ferroptosis has been implicated in experimental cardiomyopathy, but its role in primary HHD remains to be established (Fang et al., 2019).

Ferroptosis, characterized by Dixon and colleagues in 2012, is a regulated form of cell death driven by iron-dependent phospholipid peroxidation (Dixon et al., 2012). The GPX4-SLC7A11 and ACSL4-LPCAT3 axes influence antioxidant defense and ferroptosis-susceptible lipid generation, respectively (Stockwell et al., 2017; Kagan et al., 2017; Conrad et al., 2018; Jiang et al., 2021). Iron-handling proteins including TFRC and FTH1 further shape this process (Tang et al., 2021).

Ferroptosis-related pathways have been studied in both cardiovascular disease and cancer, but their direction and consequences are strongly context dependent (Fang et al., 2019; Yang et al., 2014; Wu et al., 2019; Wang et al., 2019; Tang et al., 2021). Co-occurrence of these disease areas does not establish a shared mechanism or a common treatment strategy. The pan-cancer analyses below are therefore descriptive and hypothesis generating.

The study compares a selected DCM donor with a selected non-diseased donor, uses GSE5406 as a supportive bulk-heart-failure dataset, summarizes TCGA expression scores, and reports an Ang II H9c2 mRNA experiment. These datasets are not a harmonized cross-disease cohort and are not direct evidence in HHD.

## 2 Methods

### 2.1 Data acquisition and preprocessing

Single-cell data were obtained from GSE183852 (Koenig et al., 2022). The analysis was restricted by exact sample identifiers to H_ZC-11-292 (GEO-annotated non-diseased) and HDCM1 (DCM). After the stated quality-control filters, 8,749 and 7,202 cells, respectively, were retained. H_ZC-LVAD-1 was excluded because GEO metadata classify it as dilated cardiomyopathy. Cells with fewer than 200 detected genes, mitochondrial content above 20%, or gene counts above the 99th percentile were excluded. Counts were normalized to 10,000 per cell and log1p transformed. This one-donor-per-condition design supports descriptive cell-level analysis only.

### 2.2 Single-cell transcriptomic analysis

Scanpy was used for principal-component analysis, neighborhood construction, UMAP embedding, Leiden clustering, and Wilcoxon ranking (Wolf et al., 2018). Cluster labels were assigned by overlap with curated cardiac-marker panels; unresolved clusters retained provisional marker-based labels (Franzen et al., 2019; Hu et al., 2023). Differential expression was computed globally and only within cell categories containing at least five cells from each donor. All 24,216 genes were retained in each rank result, allowing both positive and negative ranks to be evaluated. Genes with |logFC| > 0.25 and BH-adjusted p < 0.05 were labeled as cell-level contrasts. No trajectory or spatial-transcriptomic analysis was performed.

### 2.3 Ferroptosis gene set analysis

A 117-gene ferroptosis set was curated from FerrDb (Zhou and Bao, 2020); 109 genes were detected in the selected single-cell data. Scanpy score_genes produced a composite gene-expression score relative to matched control genes. Because the set combines drivers, suppressors, and markers, the score is not a direct measure of ferroptotic activity. Bulk and TCGA summaries used unweighted mean standardized expression.

### 2.4 Supportive bulk transcriptome analysis

GSE5406 contains 210 RMA-normalized human left-ventricular microarrays (Hannenhalli et al., 2006). Sample classes were assigned from GEO sample-title metadata, yielding 194 failing myocardium samples (108 ischemic and 86 idiopathic) and 16 non-failing samples. GPL96 probes were mapped to gene symbols, with the median used for genes represented by multiple probes. Welch’s t-test and Benjamini-Hochberg correction were applied; |log2FC| > 0.5 and adjusted p < 0.05 defined the combined bulk threshold.

### 2.5 Pan-cancer analysis using TCGA data

TCGA pan-cancer RNA-seq expression data covering 33 cancer types were obtained through UCSC Xena (Goldman et al., 2020). Unweighted ferroptosis-gene expression scores were summarized descriptively. Cancer lineage, tumor purity, cohort size, and expression-scale differences were not harmonized, and no patient-level model was built.

### 2.6 Donor-composition diagnostics

Cell counts and within-donor percentages were tabulated by cluster-derived category. A category was eligible for exploratory within-cell-type comparison only when at least five cells were present in each donor. Percentage-point differences are descriptive comparisons of the two selected donors and are not population estimates. Ligand-receptor and differential cell-communication analyses were not performed.

### 2.7 Statistical analysis

Analyses used Scanpy, SciPy, pandas, NumPy, gseapy, and NetworkX. Single-cell Wilcoxon rankings retained all genes and used BH correction; their p values treat cells as units and are susceptible to pseudoreplication. GSE5406 used Welch’s t-test with BH correction and the stated effect-size threshold. GO Biological Process and KEGG overrepresentation analyses used the 84 ferroptosis genes meeting the cell-level single-cell threshold (Ashburner et al., 2000; Kanehisa and Goto, 2000). Pearson correlation summarized logFC across 96 genes detected in both transcriptomic datasets. RT-qPCR comparisons used unpaired Student's t-tests, with p < 0.05 considered statistically significant; no multiplicity correction was applied across the four genes. Gene-specific cross-context results are presented in Supplementary Table S1. Statistical results are descriptive unless explicitly stated otherwise.

### 2.8 RT-qPCR mRNA assessment in H9c2 cardiomyoblasts

H9c2 rat cardiomyoblasts (Kimes and Brandt, 1976) were used as a separate Ang II-associated cellular stress model. Cells were cultured in DMEM with 10% fetal bovine serum and 1% penicillin/streptomycin and treated with Ang II (100 nM, 24 h) or vehicle. Total RNA was extracted with TRIzol, reverse transcribed using PrimeScript, and analyzed using TB Green chemistry. Expression was normalized to GAPDH using the 2^-DeltaDeltaCt method. GPX4, SLC7A11, ACSL4, and TFRC were assessed in three biological replicates.

## 3 Results

### 3.1 Single-cell transcriptomic landscape of the two selected donors

The comparison comprised H_ZC-11-292 (8,749 cells) and HDCM1 (7,202 cells), totaling 15,951 cells (Figure 1). Fourteen cluster-derived categories remained after exact sample restriction; several retained provisional marker-based labels. Cardiomyocytes (1,811 cells), macrophages (1,141), and endothelial cells (1,454) occurred only in the selected normal donor. Fibroblast, pericyte, neuronal, and lymphatic-endothelial categories contained at least five cells in both donors. All proportions and cell-level p values therefore describe these two donors and do not estimate population-level disease effects. Apparent absence from one donor may reflect sampling, quality-control, annotation, or donor effects rather than biological depletion.

**FIGURE 1:**
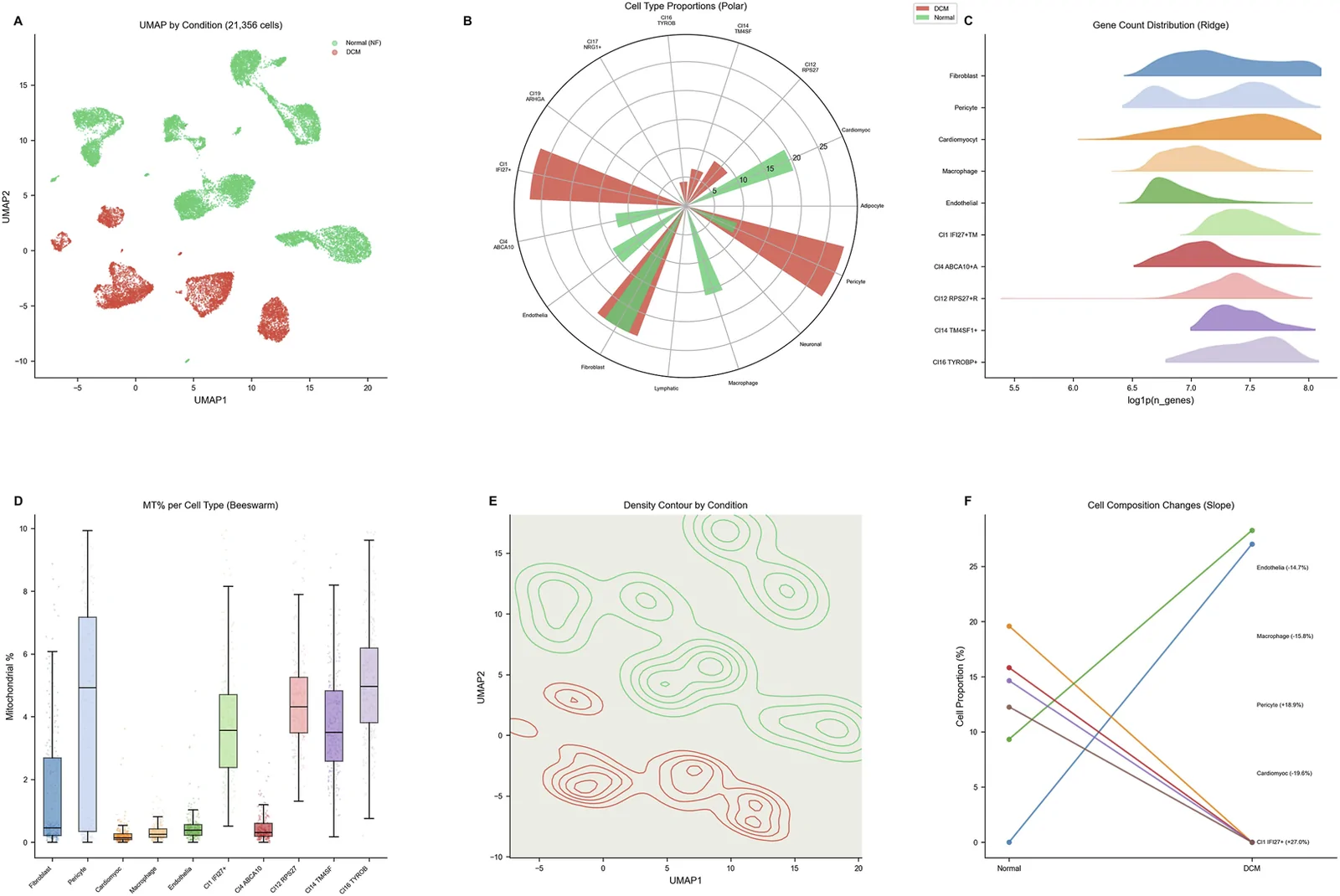
Single-cell landscape and composition of the two selected donors. **(A)** UMAP of 15,951 cells colored by selected donor condition. **(B)** Within-donor cell-category proportions. **(C,D)** Gene-count and mitochondrial-percentage quality summaries for the ten most abundant categories. **(E)** UMAP density contours. **(F)** Descriptive percentage-point differences for the six categories with the largest absolute between-donor differences. All panels compare one selected donor per condition and are not population-level disease estimates.

### 3.2 Ferroptosis gene expression atlas at single-cell resolution

Expression of 109 detected ferroptosis-related genes was summarized across the 14 cluster-derived categories (Figure 2). Condition contrasts in Figure 2B are restricted to the four categories represented in both donors. The composite score was higher in DCM cells (mean 0.289 +/− 0.071) than in normal cells (0.109 +/− 0.068), but this donor- and composition-confounded cell-level difference is not evidence of greater ferroptotic activity or a subject-level disease effect.

**FIGURE 2:**
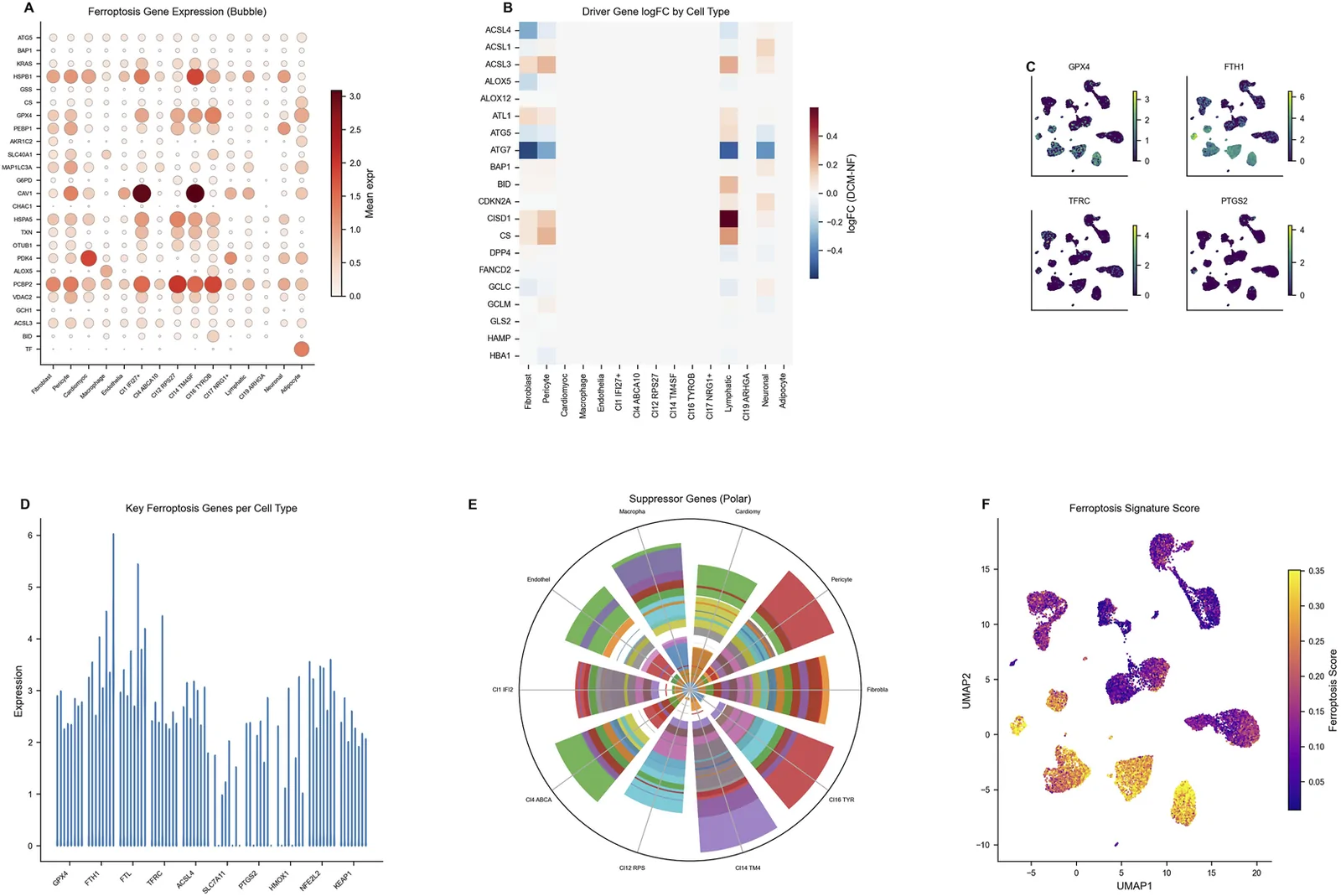
Descriptive ferroptosis-gene expression atlas at single-cell resolution. **(A)** Twenty-five selected detected genes. **(B)** Cell-level logFC for 20 selected drivers in the four shared categories. **(C)** GPX4, FTH1, TFRC, and PTGS2 UMAPs. **(D)** Mean expression of 10 selected genes in eight categories. **(E)** Within-gene-scaled means for 15 selected suppressors in ten categories. **(F)** Composite score combining drivers, suppressors, and markers; it is not a direct ferroptosis-activity measure.

### 3.3 Cell-level ferroptosis-gene contrasts and exploratory enrichment

Full-rank cell-level Wilcoxon analysis retained both ranking tails. Among 109 detected ferroptosis-related genes, 60 met the up threshold, 24 met the down threshold, and 25 were nonsignificant (Figure 3A). Large values such as ATP5MC3 (logFC = 31.03) reflect near-zero comparator expression and should not be interpreted as verified biological magnitudes. Other high positive ranks included AKR1C3 (5.10) and FTL (4.56), while PLIN4 (−5.79), CP (−5.14), and PRKAA2 (−4.38) ranked among the negative contrasts. Within the four shared cell categories, fibroblasts had 51 up and 16 down, pericytes 49 up and 4 down, neuronal cells 10 up and 1 down, and lymphatic-endothelial cells 11 up and 0 down ferroptosis-related genes (Figures 3B,E). Exploratory overrepresentation of the 84 threshold-meeting genes yielded 224 GO and 95 KEGG terms at BH-adjusted p < 0.05 (Figures 3C,D,F). All counts and enrichment inputs derive from cell-level, one-donor-per-condition tests.

**FIGURE 3:**
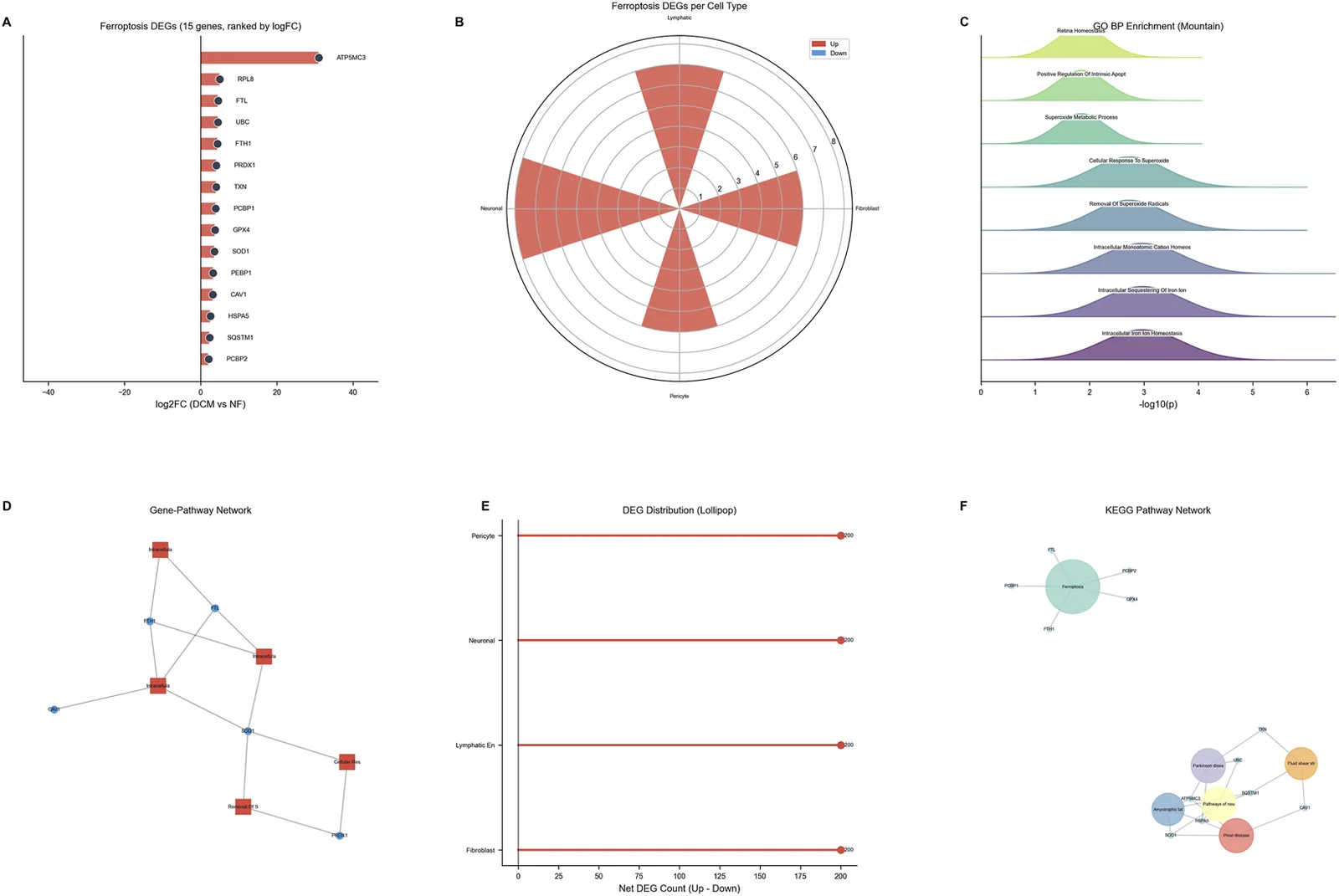
Cell-level ferroptosis-gene contrasts and exploratory enrichment. **(A)** Eight positive and eight negative full-rank cell-level contrasts. **(B)** Up/down counts in the four shared categories. **(C)** Eight GO terms shown as -log10(BH-adjusted p). **(D,F)** Four selected GO/KEGG terms and subsets of linked genes. **(E)** Net significant-gene count by shared category. P values treat cells as units and are susceptible to pseudoreplication.

### 3.4 Supportive bulk transcriptome analysis of ferroptosis-related signals

Using GEO sample-title-defined groups, 5 of 99 tested ferroptosis-related genes met |log2FC| > 0.5 and adjusted p < 0.05 (Figure 4): HBA1 was higher (log2FC = 1.278, adjusted p = 0.0279), whereas STAT3 (−0.704, 0.00248), POR (−0.630, 0.00868), TXNRD1 (−0.821, 0.0154), and SLC38A1 (−0.597, 0.0175) were lower. Across 96 ferroptosis-related genes detected in both transcriptomic datasets, fold changes were uncorrelated (Pearson r = 0.034, p = 0.740). The bulk data are supportive heart-failure context, not an HHD cohort or external validation of the single-cell result.

**FIGURE 4:**
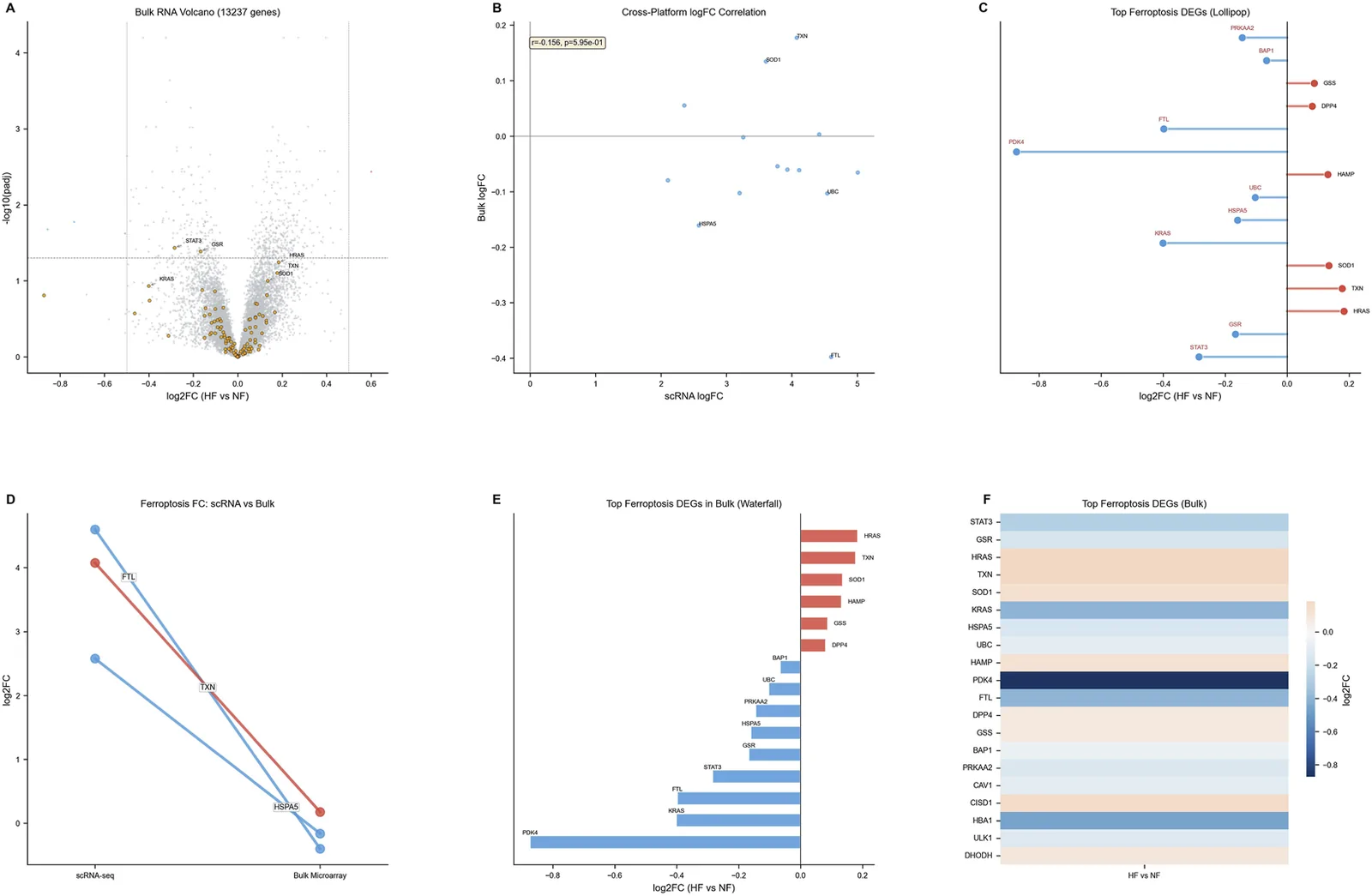
Supportive bulk microarray analysis of GSE5406. **(A)** Volcano plot after GEO-title-based assignment of 194 failing and 16 non-failing samples. **(B)** Correlation across 96 genes detected in both transcriptomic datasets. **(C,E,F)** Ferroptosis-related genes ranked by raw p value or nominal fold change. **(D)** Selected cross-platform directions. Five of 99 tested ferroptosis-related genes met |log2FC| > 0.5 and BH-adjusted p < 0.05; cross-platform correlation was absent.

### 3.5 Pan-cancer composite ferroptosis-gene expression scores

Unweighted composite scores were summarized across 33 TCGA cancer types (11,014 samples; Figure 5). Scores varied by cancer type, but the gene set combines ferroptosis drivers, suppressors, and markers; higher values cannot be interpreted as greater ferroptotic activity. The plots show score distributions, ranked means, cross-platform gene fold changes, and sample counts. No formal power analysis was performed, and lineage, tumor purity, cohort, and platform effects may explain part of the observed variation.

**FIGURE 5:**
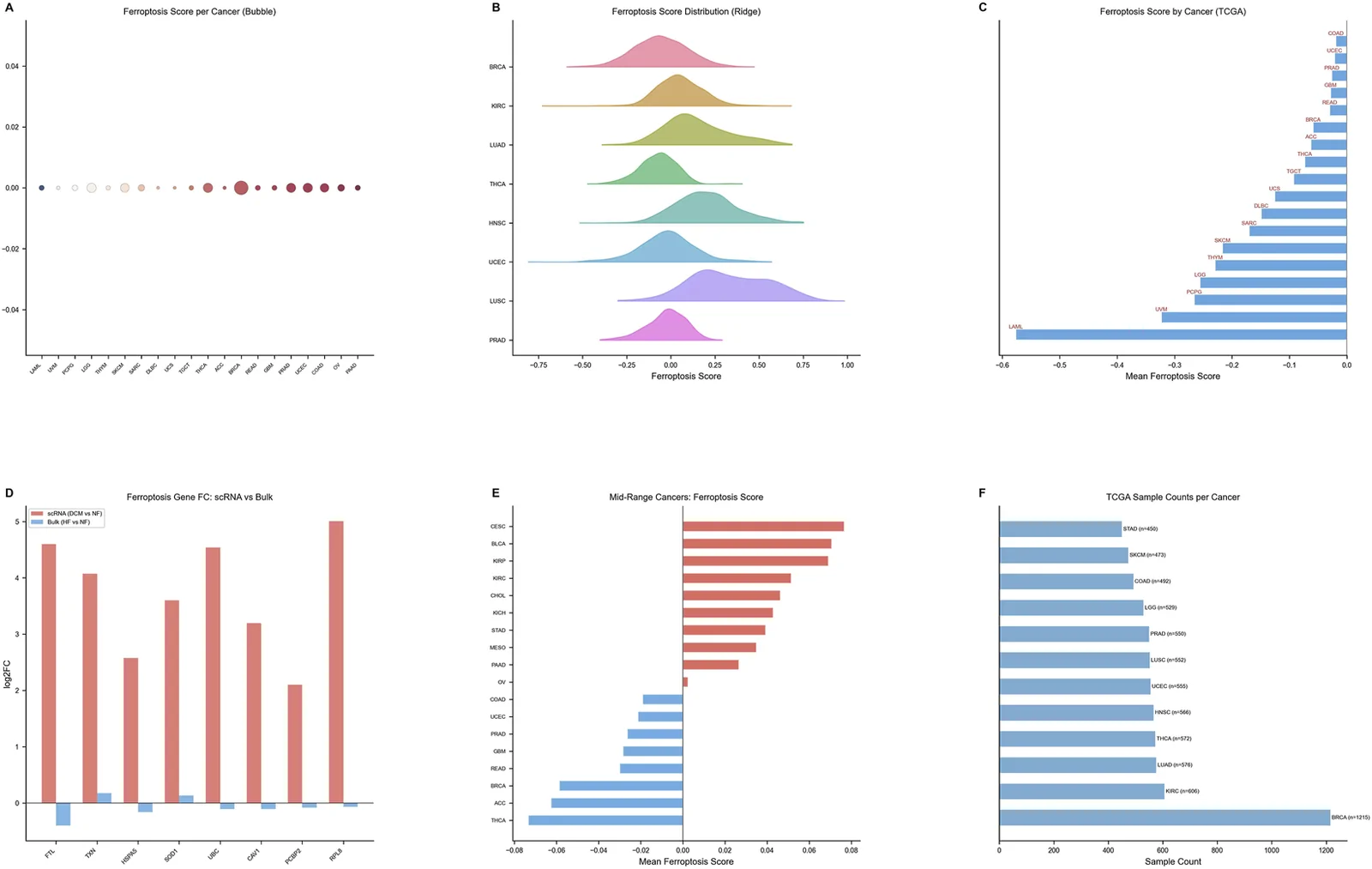
Descriptive pan-cancer composite ferroptosis-gene expression scores. **(A)** Mean composite score by cancer type; point size indicates sample count. **(B)** Score distributions for the eight largest cohorts. **(C)** Nine highest and nine lowest mean scores. **(D)** Selected single-cell and bulk logFC values. **(E)** Mid-range mean scores. **(F)** Sample counts for the 12 largest cohorts. Scores are not direct measures of ferroptotic activity, and no formal cross-cancer inference or power analysis was performed.

### 3.6 Cross-dataset contextualization

The selected single-cell dataset contained 109 detectable ferroptosis-related genes and GSE5406 contained 99 tested genes, with 96 detected in both (Figure 6A). These are detected genes, not shared DEGs. Fold-change directions varied (Figures 6B,C) and showed no cross-platform correlation (r = 0.034, p = 0.740; Figure 6E). Figure 6D displays enrichment of the significant single-cell gene list, not enrichment of the 96-gene overlap. The TCGA spectrum in Figure 6F is descriptive context only.

**FIGURE 6:**
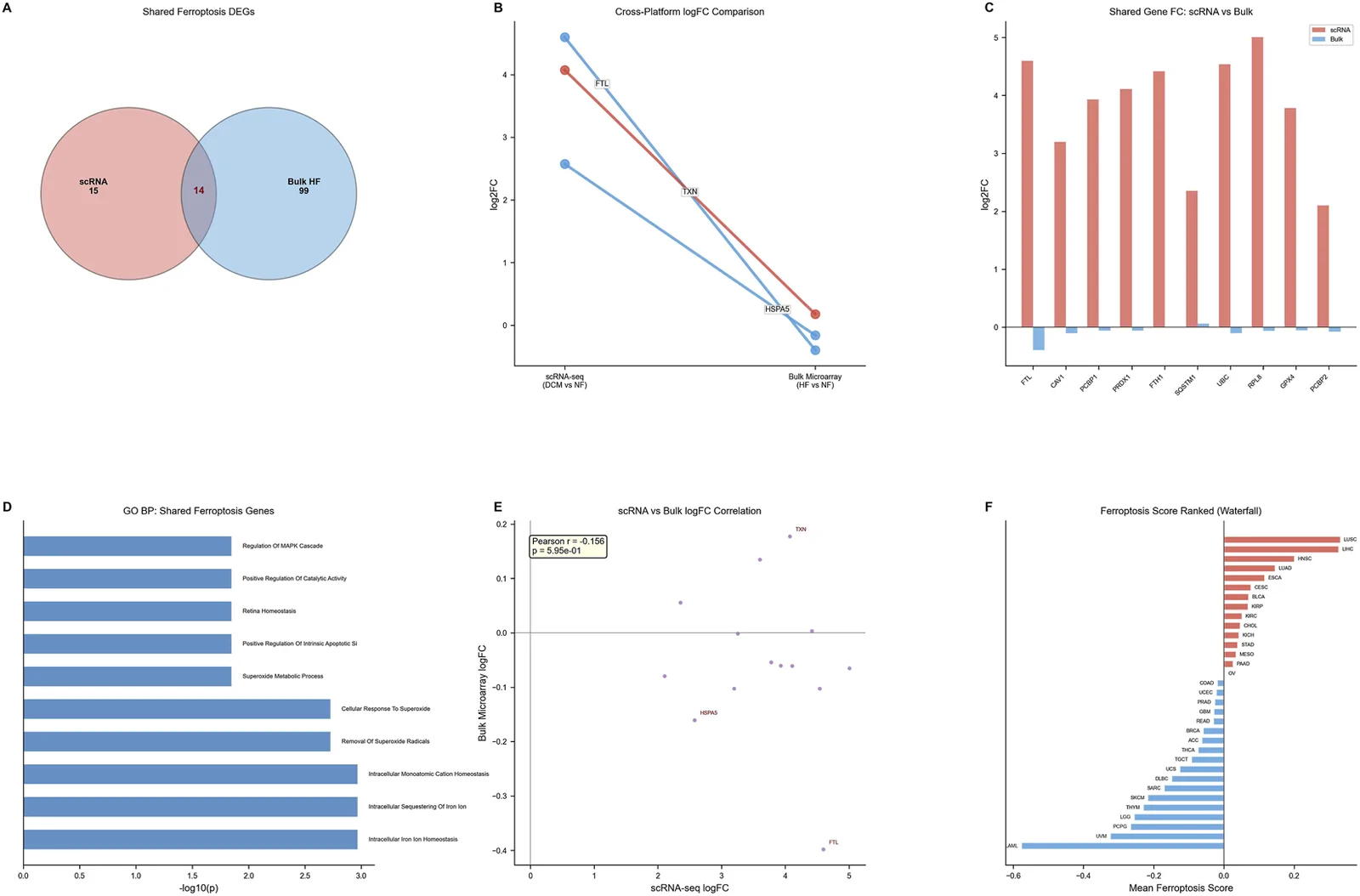
Cross-dataset contextualization of detected ferroptosis-related genes. **(A)** Overlap of 109 genes detected in the selected single-cell data and 99 tested in GSE5406; 96 were shared. These are detected genes, not shared DEGs. **(B,C)** Selected fold-change comparisons. **(D)** GO enrichment of the threshold-meeting single-cell genes, not the 96-gene overlap. **(E)** Cross-platform correlation (r = 0.034, p = 0.740). **(F)** Ranked pan-cancer composite scores.

### 3.7 Donor-composition and analysis-eligibility diagnostics

Figure 7 documents the composition imbalance underlying the inferential limits. Only four of 14 cluster-derived categories met the minimum cell-count criterion in both donors; six appeared only in the selected normal donor and four only in the selected DCM donor. Cell counts, percentages, selected-gene means, and percentage-point differences are descriptive. No immune depletion, recruitment, or altered cell-cell communication can be inferred from these two donors.

**FIGURE 7:**
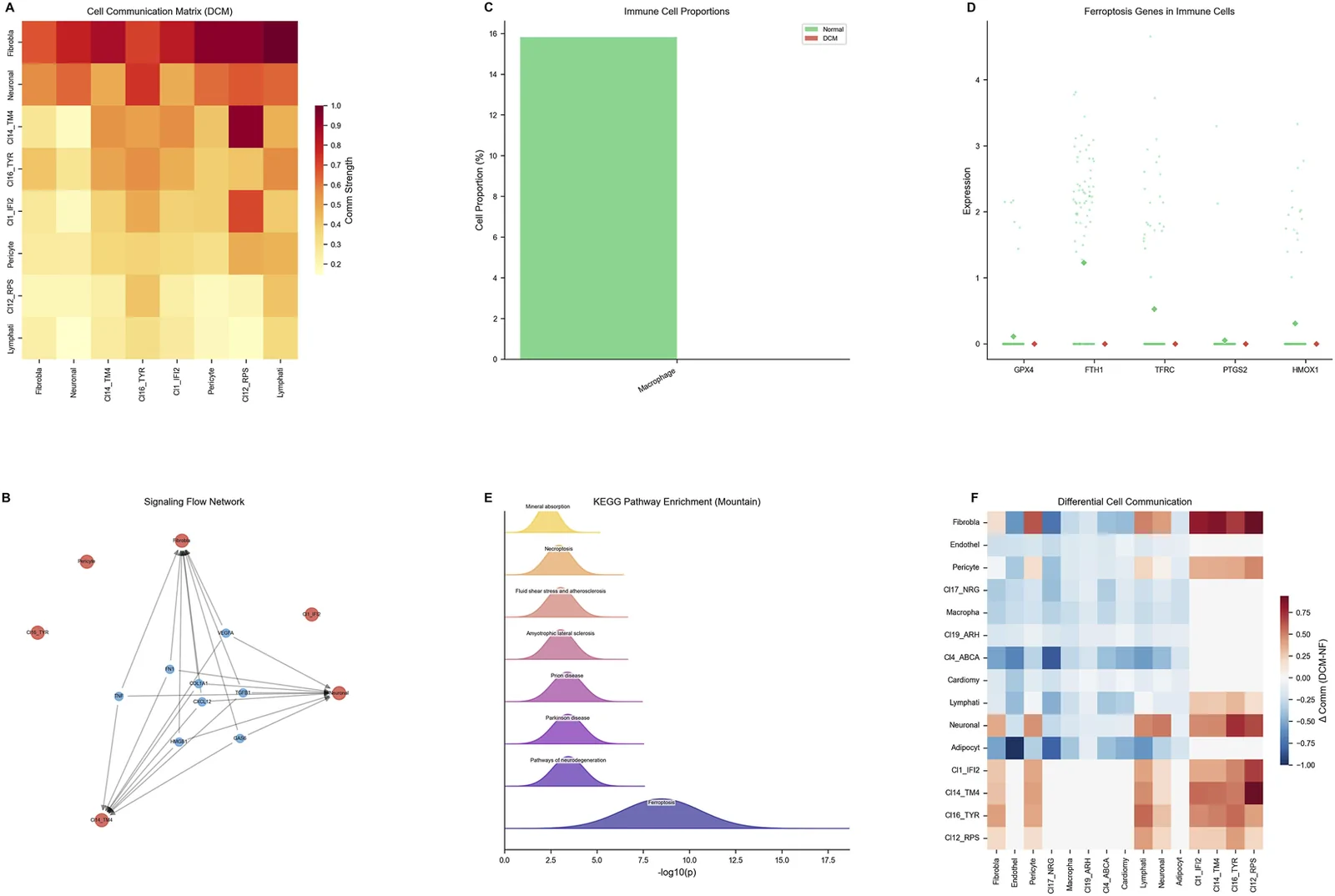
Donor-composition and analysis-eligibility diagnostics. **(A)** Cell counts for the ten most abundant categories. **(B)** Within-donor proportions for the same ten categories. **(C)** Number of categories represented in both or only one donor. **(D)** Counts in the four shared categories. **(E)** Mean normalized expression of four selected genes in shared categories. **(F)** Descriptive percentage-point differences. No population-level composition or cell-communication inference is made.

### 3.8 Cross-dataset directional summary

Figure 8 summarizes TCGA composite scores, the magnitude of single-cell contrasts, descriptive cell-level scores, GPX4 expression, and the four-gene direction comparison. GPX4 expression was higher in the selected DCM cells, but this is a composition-sensitive one-donor-per-condition observation. The final panel makes the cross-context directional differences explicit. These displays do not establish clinical validity, common biomarkers, or therapeutic applicability.

**FIGURE 8:**
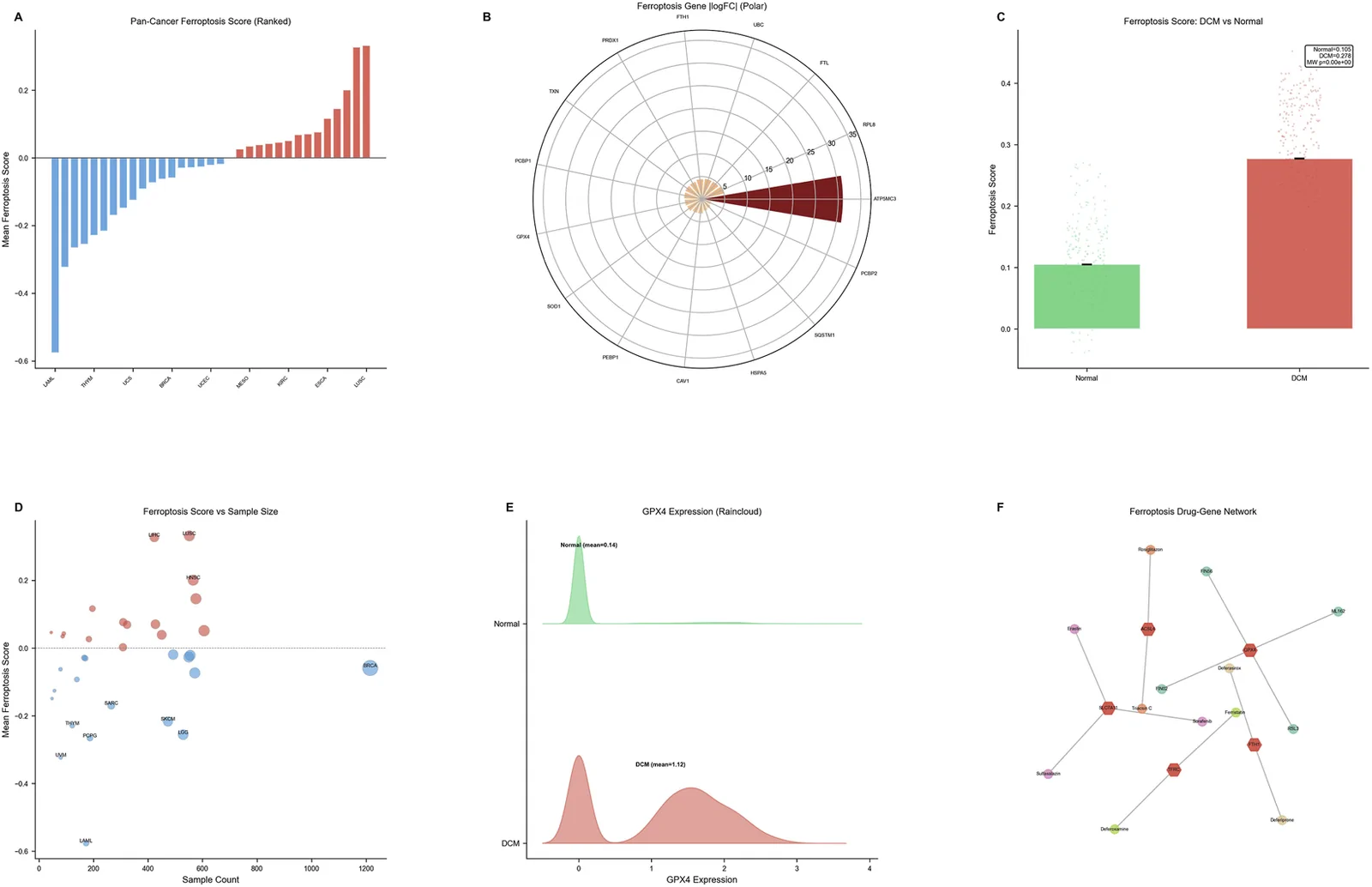
Descriptive cross-dataset summaries and four-gene direction comparison. **(A)** Ranked pan-cancer composite scores. **(B)** The 15 largest absolute single-cell logFC values. **(C)** All-cell composite-score means without donor-level uncertainty or inferential p values. **(D)** Cancer-type score versus sample count. **(E)** GPX4 expression in the selected donors. **(F)** Four-gene directions across single-cell, bulk, and RT-qPCR contexts; significance is detailed in Supplementary Table S1.

### 3.9 RT-qPCR mRNA assessment of selected genes in H9c2 cardiomyoblasts

RT-qPCR showed lower GPX4 (0.43 +/− 0.08-fold, p < 0.01) and SLC7A11 (0.51 +/− 0.11-fold, p < 0.05) and higher ACSL4 (2.34 +/− 0.31-fold, p < 0.01) and TFRC (1.87 +/− 0.24-fold, p < 0.05) after Ang II exposure (Figure 9). GPX4 was significantly positive in the cell-level single-cell comparison and nominally positive but nonsignificant in GSE5406, whereas RT-qPCR showed a decrease. ACSL4 and TFRC were significantly negative in the cell-level single-cell comparison and nominally negative but nonsignificant in GSE5406, whereas RT-qPCR showed increases. SLC7A11 was nominally negative and nonsignificant in single cells, nominally positive and nonsignificant in bulk, and lower in RT-qPCR. These data are context-dependent mRNA observations, not uniform cross-platform or functional validation (Supplementary Table S1).

**FIGURE 9:**
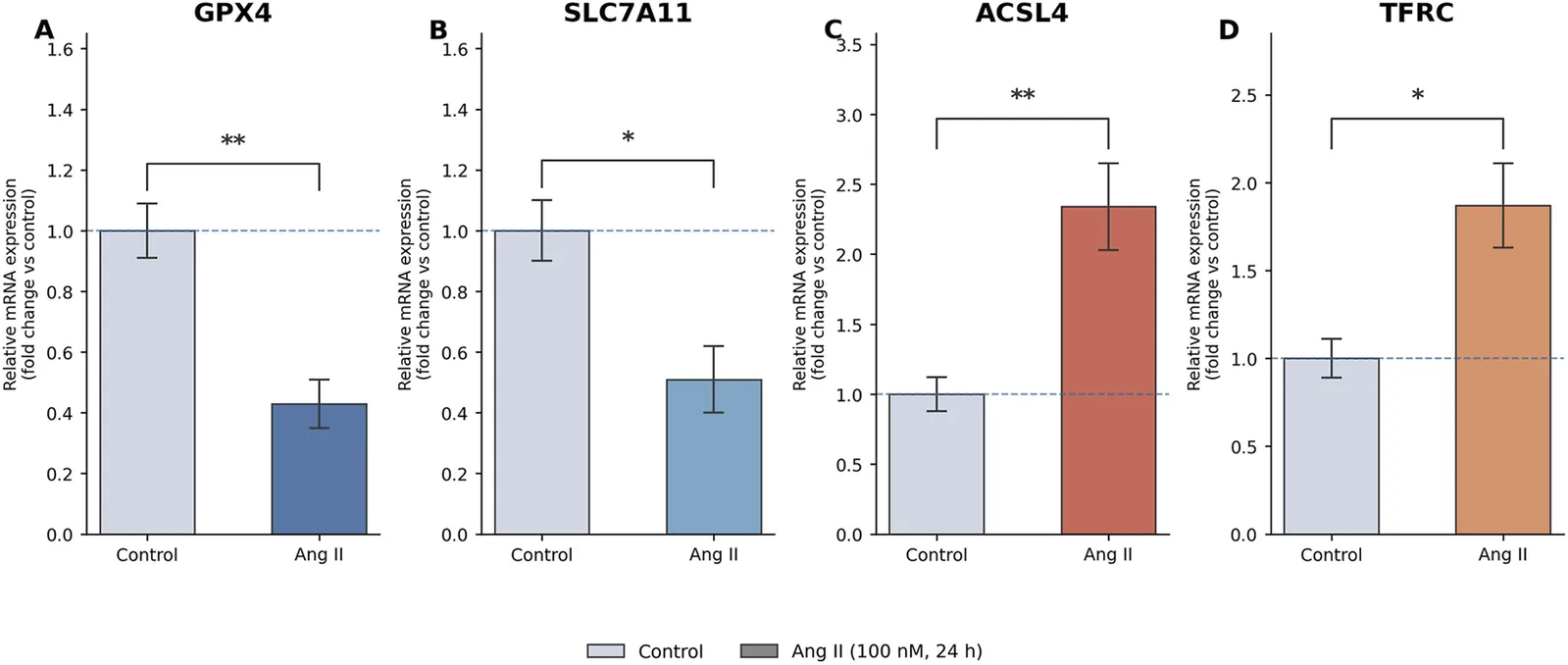
RT-qPCR assessment of selected ferroptosis-related mRNAs in Ang II-stimulated H9c2 cells. **(A–D)** GPX4, SLC7A11, ACSL4, and TFRC mRNA after Ang II versus vehicle (100 nM, 24 h); GAPDH-normalized 2^-DeltaDeltaCt, mean +/− SD, n = 3 biological replicates; *p < 0.05 and **p < 0.01 (Supplementary Table S1).

## 4 Discussion

This study is an exploratory comparison of one DCM donor and one non-diseased donor, supported by a mixed-heart-failure bulk cohort, descriptive pan-cancer summaries, and a separate H9c2 mRNA experiment. It provides neither direct HHD evidence nor a patient-level model, shared cardiac-cancer mechanism, or therapeutic claim.

Cardiomyocytes, macrophages, and endothelial cells were not detected in the selected DCM donor. Their profiles therefore cannot support condition-specific comparisons. Even for the four shared categories, cell-level statistics remain pseudoreplicated and composition sensitive. Similarities to ferroptosis biology reported in cardiovascular and tumor settings remain conceptual (Kagan et al., 2017; Fang et al., 2019; Wu et al., 2019; Wang et al., 2019).

Accession-level metadata and full-rank differential-expression outputs are essential for interpreting these comparisons. Sample identity was assigned from GEO metadata, both positive and negative ranking tails were retained, and cell-composition differences were interpreted descriptively. No cell-communication inference was performed.

Pan-cancer composite-score differences should not be read as ferroptosis activation or suppression. They may reflect lineage, purity, cohort composition, expression scale, and the mixture of drivers, suppressors, and markers in the score. No spatial transcriptomic data were analyzed; therefore, these data do not support spatial or domain-specific conclusions.

Candidate genes and pathways should be treated only as priorities for independent replication. No predictive model, AUC, or patient-level performance estimate was included, and the figures support no clinical-validation or drug-applicability conclusion.

Limitations include: (1) the single-cell comparison contains one selected non-diseased donor and one DCM donor, not primary HHD tissue; (2) cell-level tests are pseudoreplicated, and only four cluster-derived categories were represented in both donors; (3) several categories remain provisional marker-based labels; (4) GSE5406 is a mixed-heart-failure cohort and showed no cross-platform fold-change correlation across 96 detected genes; (5) the four-gene directions differed across the single-cell, bulk, and RT-qPCR settings (Supplementary Table S1); (6) the H9c2 experiment assessed mRNA expression only; protein abundance, lipid peroxidation, cell death, and rescue assays were not evaluated; (7) TCGA scores are unweighted, cross-cancer descriptive summaries; and (8) no spatial, cell-communication, predictive-model, clinical-validation, or therapeutic-efficacy conclusion is supported.

## 5 Conclusion

This exploratory study identifies donor- and context-dependent ferroptosis-related transcriptional patterns in selected DCM and non-diseased samples, a mixed-heart-failure bulk dataset, pan-cancer expression summaries, and an Ang II H9c2 mRNA model. It does not establish a causal mechanism, biomarker, or treatment target in primary HHD or cancer. Disease-specific multi-donor cohorts, transparent sample metadata, donor-level statistical designs, and protein-level and functional assays are required.

## Data Availability

The datasets presented in this study can be found in online repositories. The names of the repository/repositories and accession number(s) can be found in the article/Supplementary Material.
