# Supplementary material for: Exploratory ferroptosis-related transcriptomic patterns in dilated cardiomyopathy and pan-cancer datasets with H9c2 RT-qPCR assessment
Source: Front Genet. 2026 Sep 22;17:1909716. doi: 10.3389/fgene.2026.1909716 (PMC13638191; doi:10.3389/fgene.2026.1909716)
Supplement: Supplementary file 1 [file Table1.docx]

**Supplementary Table S1**

**Gene-specific expression directions across single-cell, bulk-transcriptome, and RT-qPCR analyses**

| **Gene** | **scRNA-seq: DCM vs Normal** | **GSE5406 bulk: HF vs NF** | **RT-qPCR: Ang II vs vehicle** | **Concordance and interpretation** |
| --- | --- | --- | --- | --- |
| **GPX4** | Up; logFC = +3.845; raw and BH-adjusted p values underflowed to 0 in the cell-level test. | Nominally up; log2FC = +0.0045; raw p = 0.942; BH-adjusted p = 0.972 (NS). | Down; 0.43 +/- 0.08-fold; p < 0.01; n = 3 biological replicates. | RT-qPCR is opposite to both transcriptomic nominal directions; the bulk change is NS. The cell-level scRNA result is not donor-level evidence. |
| **SLC7A11** | Nominally down; logFC = -1.614; raw p = 0.894; BH-adjusted p = 0.999 (NS). | Nominally up; log2FC = +0.0198; raw p = 0.597; BH-adjusted p = 0.788 (NS). | Down; 0.51 +/- 0.11-fold; p < 0.05; n = 3 biological replicates. | RT-qPCR matches the nominal scRNA direction but is opposite to the nominal bulk direction; both transcriptomic changes are NS. |
| **ACSL4** | Down; logFC = -1.453; raw p = 6.99 x 10^-23; BH-adjusted p = 3.59 x 10^-22. | Nominally down; log2FC = -0.206; raw p = 0.0356; BH-adjusted p = 0.163 (NS). | Up; 2.34 +/- 0.31-fold; p < 0.01; n = 3 biological replicates. | RT-qPCR is opposite to both transcriptomic directions; the bulk result is NS after BH correction. |
| **TFRC** | Down; logFC = -1.426; raw p = 1.08 x 10^-8; BH-adjusted p = 3.77 x 10^-8. | Nominally down; log2FC = -0.194; raw p = 0.328; BH-adjusted p = 0.578 (NS). | Up; 1.87 +/- 0.24-fold; p < 0.05; n = 3 biological replicates. | RT-qPCR is opposite to both transcriptomic directions; the bulk result is NS after BH correction. |

**Notes.** All directions are disease or stress versus the stated comparator. The single-cell comparison comprised H_ZC-11-292 (8,749 cells; non-diseased) and HDCM1 (7,202 cells; DCM). H_ZC-LVAD-1 was not included because GEO classifies it as DCM. Full-rank Wilcoxon results across 24,216 genes were used. Cell-level p values are susceptible to pseudoreplication and are not donor-level evidence. GSE5406 groups were defined from GEO sample titles (194 failing and 16 non-failing). NS, not significant after BH correction.

**Interpretive boundary.** RT-qPCR p values are unadjusted; no multiplicity correction was applied across the four tests. Differences in species, model, composition, platform, and replication unit preclude uniform cross-platform validation. RT-qPCR assesses mRNA expression and does not provide protein-level or functional ferroptosis evidence.
